# Effect of Added Sugar on the Consumption of A Lipid-Based Nutrient Supplement Among 7–24-Month-Old Children

**DOI:** 10.3390/nu12103069

**Published:** 2020-10-08

**Authors:** Harriet Okronipa, Amado D. Quezada-Sánchez, Susan L. Johnson, Cloe Rawlinson, Selene Pacheco-Miranda, Mónica Venosa López, Wendy Gonzalez Navarrete, Anabelle Bonvecchio Arenas

**Affiliations:** 1 Institute of Global Nutrition, Department of Nutrition, University of California, Davis, 3135 Meyer Hall, One Shields Avenue, Davis, CA 95616, USA; heokronipa@ucdavis.edu; 2 Department of Population Sciences and Diagnostic Medicine, Cornell University, Ithaca, NY 14850, USA; 3 Center for Evaluation and Surveys Research, National Institute of Public Health, Avenida Universidad, Santa María Ahuacatitlán, Cuernavaca 62100, Mexico; amado.quezada@insp.mx; 4 Department of Pediatrics, Section of Nutrition, School of Medicine, University of Colorado Anschutz Medical Campus, East 17th Place Mail Stop C225, Aurora, CO 80045, USA; susan.johnson@cuanschutz.edu; 5 Center for Research in Nutrition and Health, National Institute of Public Health, Avenida Universidad 655, Santa María Ahuacatitlán, Cuernavaca 62100, Mexico; clj.rawlinson@gmail.com (C.R.); selene.86517@gmail.com (S.P.-M.); venosa.lm@gmail.com (M.V.L.); 6 Global Alliance for Improved Nutrition, Rue de Vermont 37–39, 1202 Genève, Switzerland; wgonzalez@gainhealth.org; 7 Cerrada de Fray Pedro de Gante 50, Sección XVI, Tlalpan, Mexico City 14080, Mexico

**Keywords:** small quantity lipid-based nutrient supplements, acceptability, malnutrition, Mexico

## Abstract

Small-quantity lipid-based nutrient supplements (SQ-LNS) could help prevent malnutrition. Our primary objective was to examine the acceptability and consumption of sweetened and unsweetened versions of SQ-LNS before and after 14-days of repeated exposure. A total of 78 mother-infant dyads recruited from health centers in Morelos, Mexico, were randomized to two groups of SQ-LNS (sweetened, LNS-S; unsweetened, LNS-U). During the study, infants were fed SQ-LNS (20 g) mixed with 30 g of complementary food of the caregiver’s choice. The amount of supplement-food mixture consumed was measured before, during and after a 14-day home exposure period. We defined acceptability as consumption of at least 50% of the offered food mixture. At initial exposure, LNS-U consumption was on average 44.0% (95% CI: 31.4, 58.5) and LNS-S 34.8% (25.3, 44.0); at final exposure, LNS-U and LNS-S consumption were 38.5% (27.8, 54.0) and 31.5% (21.6, 43.0). The average change in consumption did not differ between the groups (2.2 p.p. (−17.2, 24.4)). We conclude that the acceptability of sweetened and unsweetened SQ-LNS was low in this study population. Since consumption did not differ between supplement versions, we encourage the use of the unsweetened version given the potential effects that added sugar may have on weight gain especially in regions facing the double burden of malnutrition.

## 1. Introduction

Malnutrition remains a public health problem globally. Over the years, several strategies to address malnutrition have been described [1,2]. Small-quantity lipid-based nutrient supplements (SQ-LNS), may have the potential to improve growth and development in children [3,4,5,6] and to prevent malnutrition by enriching the diets of children through home fortification of meals [3,7,8,9,10,11,12]. SQ-LNS products are slightly sweetened with the aim of increasing palatability and have been reported to be acceptable in different countries [13,14,15]. Whether the added sweetening agent improves palatability and acceptance for young children has not been rigorously tested to date.

Many countries, including Mexico, are currently undergoing a nutrition transition where obesity rates are on the increase [16,17]. Current complementary feeding recommendations include avoiding the intake of foods with added sugar [18]. Examining whether SQ-LNS without added sugar performs similarly to the sweetened SQ-LNS is important especially in countries like Mexico where obesity is a growing problem.

Taste plays an important role in food consumption. Whereas children are born with a preference for sweet taste [19,20], they also have the ability to learn to like new flavors through repeated dietary exposure; though it may require as many as 8–15 exposures [21,22,23,24,25] before infants learn to like new foods [26]. Thus, repeated experiences with a novel food, such as SQ-LNS, might be effective in increasing acceptability and intake over time [27]. To the best of our knowledge, no studies have been designed to examine this principle of repeated exposure using LNS products. We designed the present study to: (1) examine and compare the acceptability and consumption of a sweetened and unsweetened version of SQ-LNS; (2) examine the consumption patterns of the supplements during a two-week home exposure period; and (3) examine if repeated exposure to the supplement would impact consumption. We specified two main research hypotheses: (1) Average percentage of food mixture consumed will increase from initial to final exposure in both treatment groups. (2) Average change of percentage of food mixture consumed will differ between treatment groups.

## 2. Materials and Methods

### 2.1. Study Setting, Design and Participants

The palatability study (March-June 2020) was conducted in semi-urban communities located in the State of Morelos, in south-central Mexico. The study was designed as a randomized field trial involving two treatment groups—one randomized to sweetened SQ-LNS (LNS-S) and the other randomized to unsweetened SQ-LNS (LNS-U).

Caregivers and their children were recruited from 6 health centers from 4 municipalities (Emiliano Zapata, Xochitepec, Temixco and Coatetelco) in the study area from March to May 2019. At the health centers, caregivers were invited for screening if they had children aged between 7 and 24 months. Caregivers were considered eligible for the study if they (1) were aged 18 years or more, (2) were the main person who fed the child, (3) did not have a history of food allergy, (4) could read and write, (5) lived in the study area and (6) were not a beneficiary of programs that provided other micronutrient supplements at the time of the study such as PROSPERA (a national conditional cash transfer program in Mexico designed to alleviate poverty through cash transfers that were conditioned upon compliance with specific services aimed at improving health, nutrition, and education.

Children were considered eligible if they (1) were aged between 7 and 24 months, (2) were born at term (gestation period >37 weeks), (3) had begun complementary feeding, (4) had been exposed to nuts (including peanuts), (5) did not have any food allergy or intolerance and (6) did not have any reported illnesses or metabolic disorders that could affect food intake.

Written informed consent was obtained from all eligible caregivers who agreed to be part of the study. The project study was conducted following the rules of the Declaration of Helsinki of 1975, revised in 2013. The study was approved by the Institutional Review Boards of the National Institute of Public Health (Ethics Research—ID-approval CI-1581, Biosafety and Research Committees), Mexico and of the University of California, Davis—ID-approval 1298170–3.

### 2.2. SQ-LNS Products

The SQ-LNS products used in this study were developed and supplied by Nutriset (Malaunay, France). The supplements were packaged in 20 g sachets and labelled specifically for this study (SQ-LNS infant-A; SQ-LNS infant-B). The supplements contained vegetable oil, skimmed milk powder, peanuts, maltodextrin and a vitamin/mineral premix and provided about 118 kcal of energy (Figure S1: SQ-LNS nutritional information). The sweetened version (standard version), included 1.6 g of added sugar/sachet.

### 2.3. Randomization and Blinding

After being recruited by field workers, participants were randomly assigned into one of two groups using a computer-generated scheme (SAS version 9.3, SAS Institute, Car, NC, USA) in blocks of 4 and 8, prepared by a statistician who was not a part of the study team. To ensure a balanced sample size per age group, a separate randomization list for each of the two age groups (7–12 months, 13–24 months) was created. These two age groups were constructed to account for developmental differences in children’s eating and feeding skills. The randomization process was done by a supervisor and an assistant. Sheets bearing supplement allocations represented by the two different inscriptions “SQ-LNS Group A” and “SQ-LNS Group B” (based on the randomization list) and numbered from 1 to 40, separately for each age group (7 to 12 months and 13 to 24 months), were created. Each sheet was then placed in an opaque envelope (numbered 1 to 40 for each age group) and stacked in ascending order. For each randomization, the supervisor picked up the first 8 topmost envelopes in the stack and shuffled them, after which the assistant picked one to reveal the allocation. Allocation information was kept by the supervisor in a password-protected file.

The two versions of the supplement were identically packaged in sachets and were differentiated by a small inscription of the letter’s “A” or “B” on each supplement sachet. Except for the project coordinator, all data collectors, study investigators and participants were blinded to the assigned treatment group. All investigators involved in data analysis remained blinded until the initial analysis of primary outcomes was completed.

### 2.4. Study Procedures

Three home visits were carried out during the study period. At the first home visit (herein referred to as initial exposure visit), caregivers were asked not to not feed their child during the hour prior to the team’s arrival time. Caregivers were requested to provide 30 g (~2 tablespoons) of a food commonly fed to their children, into which one sachet (20 g) of the assigned version of SQ-LNS was mixed and weighed. We decided to have caregivers mix the supplement with complementary food of their choice because this was a field trial and we wanted to have the experimental conditions be as ecologically valid (close to the real world) as possible. Additionally, in Mexico there is not a universal complementary food consumed by most children, unlike in other countries, such as some countries in Africa where maize porridge is a common complementary food fed to infants. A similar additional preparation was made, this time using one sachet of the non-assigned SQ-LNS version. Field workers offered bananas in the case that the caregiver did not have any food ready or available. Caregivers were requested to first feed two bites of the mixture containing the non-assigned SQ-LNS version, followed by unlimited bites of the mixture containing the assigned SQ-LNS. They were instructed to feed the food mixture to the child (in the usual feeding position, pace and style they normally fed the child) until s/he refused it 3 consecutive times or until they thought the child was full, whichever came first. At the end of the feeding session, the amount of food mixture consumed was calculated by subtracting the weight of the container + leftover food remaining from the weight of the container + food mixture prior to feeding.

At the end of the initial visit, caregivers received a home exposure package containing 14 sachets of SQ-LNS (assigned version), 14 pre-weighed feeding containers with lids (labelled #1 to #14, for Day 1 to Day 14 of the home exposure period), a 30 mL measuring spoon, and a printed food diary to record daily information. We also provided a calendar with instructions on how to use the supplement, possible side effects and what to do when the child became sick while consuming the supplement. Caregivers were instructed to feed one sachet per day to the child for the next 14 days [13,14,15,28] by measuring 1 serving (one scoop of the 30 mL spoon) of complementary food of their choice into the feeding container provided and then thoroughly mixing in one sachet of SQ-LNS. Once the child was finished eating, caregivers were instructed to leave all leftovers in the feeding container and store them in the refrigerator or freezer. Caregivers were requested to fill out the food diary after each feeding of the supplement with information including how the supplement was fed to the child (alone or mixed with food), the type of food used, the overall feeding experience, as well as report their perception of the amount consumed by the child compared to usual intake.

Field workers conducted an intermediary home visit 7 days after the first home visit to pick up and weigh all leftovers and all used and unused supplement sachets for the first week (Days 1 to 6 of the home exposure period). In addition, they checked the food diary for Days 1 to 6, resolved doubts and concerns of caregivers, and administered study questionnaires.

At the end of the 14-day home exposure period, the study team conducted a third home visit (herein referred to as final visit) during which a second observed feeding session was carried out, following the same procedures as the first home visit. At the end of the feeding session, the study team collected and weighed all remaining leftovers as well as all used and unused supplement sachets for the second week (Days 7 to 14 of the home exposure period). The daily amount of food mixture consumed during the home exposure was calculated by subtracting the left-over mixture for a particular day from the initial weight of food mixture. The latter was estimated based on information provided in the food diary about the complementary food used to mix the supplements for each day. For each of the reported foods, the study team purchased or prepared the food as described in the food diary and one serving (1 scoop of the 30 mL spoon) was weighed on a food scale (to the nearest 0.1 g) and recorded.

### 2.5. Measures

Data were collected using research electronic data capture (REDCap) software [29] (licensed by the National Institute of Public Health, Mexico) on portable computers (tablets) and cellphones. Questionnaires (previously tested during a pilot study) were programmed and administered to participants in Spanish. Prior to data collection, field workers were extensively trained on interview skills, the content of each questionnaire, and on the use of the REDCap using lectures, role-play and discussions. They were also trained and standardized to conduct caregiver and child anthropometric measurements.

During recruitment, we collected data on maternal and child characteristics (to screen potential participants) using standard questionnaires. Child date of birth and age were recorded based on parental reports; children were categorized into two groups (7–12 and 13–24 months).

During the initial exposure visit, field workers assessed household food insecurity using the Latin American and Caribbean Food Security Scale (ELCSA), a 15-item scale (7 items for households with children) that asks respondents whether they or any other household member has experienced a certain manifestation of food insecurity in the previous three months. Response to each item was coded as yes (1) or no (0). Total scores on the scale ranged from 0 to 8 (for households without children) or 0 to 15 (for households with children). Households were classified as food secure (score 0), mildly food insecure (score 1–5), moderately food insecure (score 6–10) or severely food insecure (score 11–15) based on their total scores. Information on household socio-demographic characteristics was collected using questionnaires.

Anthropometric measurements (weight, length/height) of children and their caregivers were taken during the intermediate visit by trained and standardized field workers. The measurements were carried out in duplicate to ensure accuracy. A third measurement was taken when the difference between the first and second measurement was more than 0.1 kg for weight or 0.5 cm for length/height. Child recumbent length and caregiver height were measured using a wooden stadiometer (Shorr Productions, Olney, MD, USA); caregiver and child weight were measured using a TANITA scale (Model 1582, USA). The average values of all measurements taken were used in the analysis. Caregiver body mass index (BMI) was calculated (kg/m^2^), and dichotomous variables were created for underweight (BMI < 18.5), normal weight (18.5 ≤ BMI < 25), overweight (25 ≤ BMI < 30) and obesity (BMI > 30). Child length-for-age z-score (LAZ), weight-for-length z-score (WLZ) and weight-for-age z-score (WAZ) were calculated using the WHO ANTHRO program [30]. Dichotomous variables were created for stunting (LAZ < −2), wasting (WLZ < −2), underweight (WAZ < −2) and overweight (WLZ > + 2).

Child morbidity was monitored weekly (at each home visit), based on maternal report of the occurrence of various symptoms during the past 7 days preceding the home visit (diarrhea, fever, cough, vomiting, nasal discharge, difficulty in breathing, rapid breathing, and other symptoms). For the initial and final visits, the visit was rescheduled if the caregiver reported child illness that in their opinion influenced the child’s appetite.

To estimate the percentage of food mixture consumed, we divided the weight of the food mixture consumed by the estimated initial weight of the prepared food mixture prior to feeding. Consumption was defined as acceptable if the mean consumption of the food mixture was greater than or equal to 50% of the amount offered [11,13,14,15]. Additionally, the proportion of children who consumed more than 50% of the food mixture that was offered was examined.

### 2.6. Sample Size Calculation

A sample size of 80 was initially planned for the palatability study. For the present analysis, we had complete data for 56 participants. Considering a standard deviation of 34 percentage points (p.p.) based on a pilot study conducted in the area, a correlation of 0.3 between initial and final measurements, and a sample size of *n* = 28 for each group, our study had a 80% power to detect an average change of 18.0 p.p. in mean percent of food mixture consumed within groups and a difference in differences of 30.0 p.p.

### 2.7. Statistical Analysis

General characteristics of participants including household food security and child morbidity symptoms were examined using descriptive statistics; means ± SD were calculated for continuous variables and percentages for categorical variables.

#### 2.7.1. Analysis of Initial and Final Exposure Data

To estimate mean percentage consumption of food mixture at initial and final exposure for both study groups and their changes, we fit a fractional logistic regression [31] which is a generalization of logistic regression for outcomes that vary between 0 and 1 in a continuous scale. The fraction of food mixture consumed was specified as the outcome variable. The linear predictor included an indicator variable of study group (0 = LNS-U, 1 = LNS-S), and indicator variable of study stage (0 = initial, 1 = final), and their interaction. Percentage of supplement consumed was calculated by applying the inverse of the logit function to the linear predictor. An additional model was estimated including unbalanced variables between study groups as predictors. After model estimation, predictive margins of percentage consumed at each stage (initial or final) and each study group were obtained, in order to adjust for covariate distributions between study groups so that both groups share the same distribution of covariates. Study groups were compared at both initial and final exposure, as well as their changes, by calculating bootstrap bias corrected 95% confidence intervals with 1000 replicates. For each iteration, participants were resampled from each study group.

For the analysis of the percentage of subjects who consumed at least 50% of the supplement, logistic regression models were specified with the same linear predictors as described above. Predictive margins and comparisons between groups were obtained in an analogous way.

#### 2.7.2. Analysis of Consumption during the Home Exposure Period

To identify different patterns of consumption during the home exposure period, we performed a hierarchical cluster analysis, which is an exploratory technique for grouping observations that share similar data patterns across a set of variables. Since this statistical method requires complete data for each subject, we identified and imputed missing values for some data points to increase the analysis sample size. A total of 41 (59.3%) out of 56 subjects had complete data from the home exposure period based on completed food dairies. For each missing data point, we calculated the mean of the values from the immediate neighbors (i.e., the value of the immediate day before and the immediate day after). Daily consumption of cases with two or more missing consecutive days were not imputed. A total of 12 (2.8%) out of 658 days-person were imputed resulting in a total sample size of 47 subjects with complete data for the 14-days of home exposure.

The hierarchical cluster analysis was performed on data profiles from the percent of food mixture consumed along the 14-day home-exposure period, including the initial and final measurements, to group subjects according to their similarities. The Euclidean distance between the farthest neighbors was used as a metric of dissimilarity between groups. The number of groups was determined using a dendrogram. Mean profiles and individual profiles were plotted for each group. Each group was labeled according to the main characteristic of its mean profile. Individual profiles of subjects were plotted for each group, separately. The distribution of observations over the profile groups were compared between study groups using a contingency table and a test of homogeneity in distribution was performed using Fisher’s exact test.

Data were analyzed using SAS version 9.3 (SAS Institute, Cary, NC, USA) and Stata version 16.0 (StataCorp LLC, TX, USA). Statistical significance was set at the 0.05 level. Research hypotheses were specified data collection. The analysis plan was pre-specified, and any data-driven analyses were clearly identified as exploratory and discussed appropriately.

## 3. Results

### 3.1. Participants

**Figure 1:**
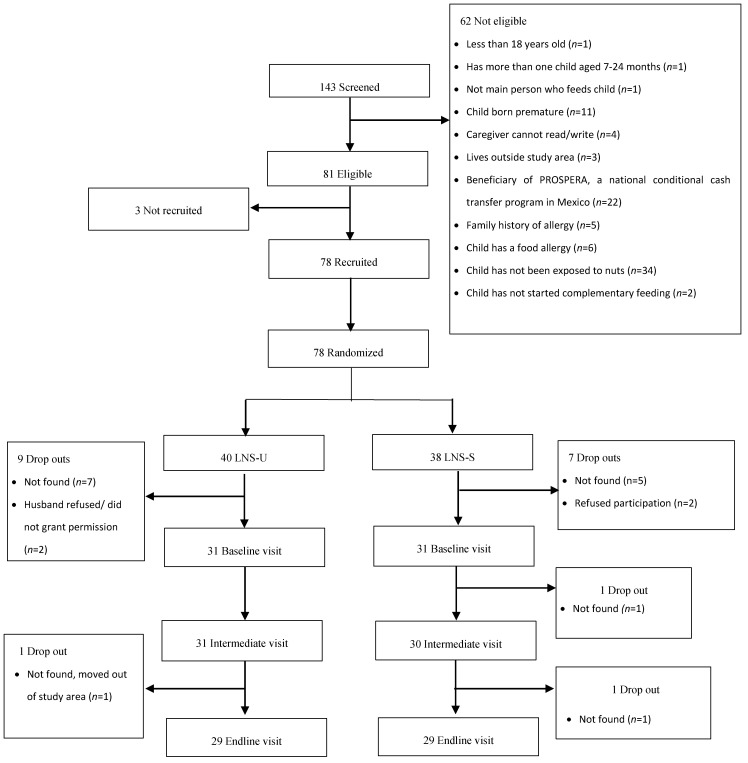
Study profile. In the unsweetened small-quantity lipid-based nutrient supplements group (LNS-U), children were randomized to receive 20 g of unsweetened small-quantity lipid-based nutrient supplements; in the sweetened small-quantity lipid-based nutrient supplements group (LNS-S), children were randomized to receive 20 g of sweetened small-quantity lipid-based nutrient supplements. For both supplement versions, children were fed their assigned version of the supplement mixed with 30 g of a complementary food of the caregiver’s choice during an observed feeding session; and one daily serving of a 30 mL spoon of a complementary food of the caregiver’s choice during a 14-day home exposure session.

The study profile is shown in Figure 1. A total of 143 women were screened for eligibility. Of these, 62 were excluded mainly due to the child’s lack of previous exposure to nuts (*n* = 34), caregiver or a member of her family being a beneficiary of PROSPERA (*n* = 22), reported food allergy or family history of allergies (*n* = 11) and/or child prematurity (*n* = 11). Of the 81 eligible women, 3 were not recruited because they either declined to participate (*n* = 2) or reported that their husbands did not like visitors coming into their home. Women (*n* = 78) were recruited (signed informed consent) and randomly assigned to SQ-LNS groups; 40 to LNS-U group and 38 to the LNS-S group. Of these, 62 completed the initial exposure visit and 59 completed the final exposure visit (LNS-U, 30; LNS-S, 29). A total of 56 had complete information on food mixture consumption at initial and final exposure and were included in the present analysis. The reasons for loss to follow-up during the study are detailed in Figure 1.

Maternal, child and household characteristics are reported in Table 1. On average, caregivers were in their mid-twenties, had an average of 2 children and were mostly stay-at-home mothers. About 25% had completed high school or a higher level of formal education. Approximately a third (32%) of caregivers were overweight and 23% were obese; only 2 caregivers were underweight.

The average child age was 14.1 ± 5.0 months; 46% were aged 7–12 months and 54% were aged 13–24 months (42% male). Four children were stunted, one met wasting criteria and 2 were underweight. None of the children were overweight or obese.

All households had electricity and over 90% had access to pipe-borne water; more than 80% of women reported bottled water as their main source of drinking water. About 80% of women (*n* = 44) lived in households experiencing some level of food insecurity. Of those (*n* = 44) who lived in food-insecure households, more than half (*n* = 28, 64%) were mildly food insecure, about a third of them (*n* = 13, 30%) were moderately food insecure and about 7% (*n* = 3) were severely food insecure.

There were few differences between the demographic characteristics of both SQ-LNS groups. Children in the LNS-U group were more likely to be male (58% vs. 26%) and had higher averages of length-for-age and weight-for-age z-scores compared to the LNS-S group.

### 3.2. Acceptability of SQ-LNS

#### 3.2.1. Consumption during Initial and Final Exposure

During the initial exposure visit, all participants in both groups mixed the supplement with food before feeding to the child. At the final visit however, about a fifth (*n* = 12) of caregivers fed the supplement alone without mixing it with food (LNS-U, *n* = 4; LNS-S, *n* = 8). At both time points, more than 60% of caregivers preferred to mix the supplement with banana (provided by the study team). Other foods commonly used included sweetened yoghurt, “petite suisse” (a sweet dairy product designed for kids), fruit, broth, soup and rice. When foods were categorized into sweet and savory groups, we observed that at both time points, more than 80% (first exposure, 83%; final exposure, 95%) mixed the supplement with foods that were sweet in taste.

At initial exposure, children in the LNS-U group consumed on average 44.0% (95% CI: 31.4, 58.5) of the food mixture whilst children in the LNS-S group consumed 34.8% (25.3, 44.0). At final exposure, LNS-U consumption was on average 38.5% (27.8, 54.0) compared to 31.5% (21.6, 43.0) of LNS-S. Average consumption did not significantly differ between study groups (*p* > 0.250), nor did average change in consumption differ (*p* = 0.837; Table 2).

After adjusting for unbalanced covariates (child sex, LAZ and WAZ), point estimates of average percentage of food mixture consumed were closer between study groups and their differences remained non-significant. Consumption did not differ by child age group at both initial and final exposure (*p* > 0.2). The number of children with morbidity symptoms the week before the interview at initial and final exposure were not significantly different between the two groups (Table S1: frequency of morbidity symptoms (during the week before the interview) of the participants, by study group and study stage). Having a cough or mucus the week preceding the interview was associated with lower consumption (−12.3 ± 5.8 p.p., *p* = 0.035) but results by study group and study stage were maintained and similar to the unadjusted estimates.

There were no differences between study groups in the percentage of children who consumed the criterion amount of the food mixture (≧50%; Table 3). At both initial and final exposure, less than half of the children in the sample consumed at least 50% of the food mixture in both LNS-U and LNS-S groups.

#### 3.2.2. Consumption during the Home Exposure Period

We used the number of empty supplement sachets collected over the 14-day period as a proxy for the total number of days the supplement was consumed. On average, children consumed the supplement on 12.3 out of 14 days (Week 1, 6.3 ± 1.1 days; Week 2, 6.0 ± 1.5). The number of days that the supplement was consumed over the exposure period did not significantly vary between the two groups. Children consumed an average of 52.9% ± 33.8 of the prepared daily food mixture.

Eighty percent of the time, caregivers mixed the supplement with a food. They used a variety of foods, which differed across days over the 14-day exposure period (range = 1 to 12 different foods over the 14-day period). Banana was most often used followed by a variety of other fruits and different flavors of sweetened yoghurt. Rice, beans, soups and broths were also used though less frequently.

**Figure 2:**
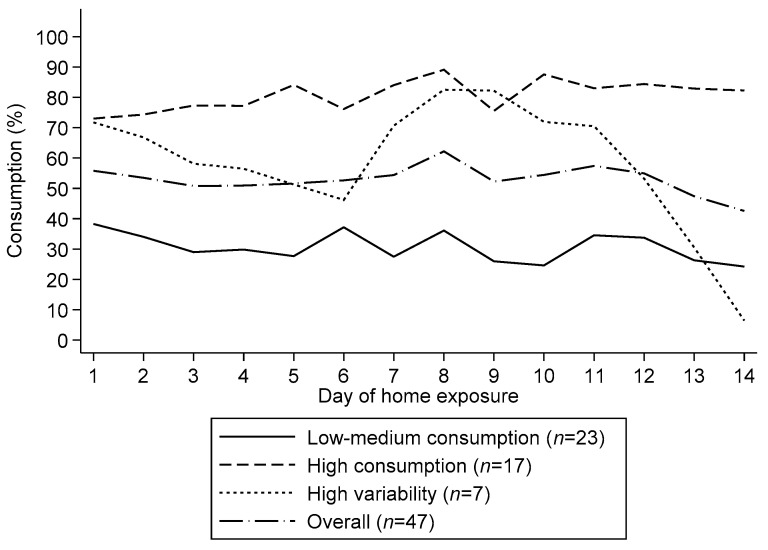
Children’s mean consumption patterns of small quantity lipid-based nutrient supplements during a 14-day home exposure period. We identified three consumption groups namely low-medium consumption, high consumption and high variability.

From the hierarchical cluster analysis, we identified 3 consumption patterns during the 2-week exposure: 1) low to medium consumption, 2) high consumption, and 3) high variability in consumption (Figure 2). Considering all 658 data points (47 children with complete data x 14 days of exposure), the median consumption (P25, P75) in the low to medium and high consumption group was 30.0% (10.0, 48.0) and 88.0% (70.6, 97.3), respectively. The third group, which we called the high variability group, had a median consumption of 68.3% (25.0, 92.2). The distribution of these patterns did not differ between study groups (*p* = 0.529) neither did they differ by child age group (*p* = 0.849). Individual profiles by profile group are shown as supplementary material (Figure S2. Individual profiles of children’s supplement consumption by consumption group).

## 4. Discussion

This study aimed to examine whether the unsweetened and sweetened versions of the SQ-LNS offered to young children differed in acceptability. Like previous studies [13,14,15,28], we defined acceptability as consumption of 50% or more of the amount offered during the observed home feeding session. Our results suggest that in this population of 7–24 months old Mexican infants and children, the acceptability of both versions of the supplement was low. Consumption did not vary significantly by supplement version nor by child age group. Additionally, we identified three distinct consumption patterns that emerged during the 14-day home exposure period. Lastly, consumption did not change after a 14-day period of repeated exposure.

Our results are different from those of other studies that have reported high acceptability of SQ-LNS (sweetened version) among children [13,14,15,28]. Several factors may explain the differences in results. First, there was low exposure to peanuts in our study area. In all of the African countries (Ghana, Burkina Faso and Malawi) where these acceptability studies were conducted, peanuts are commonly consumed. Generally, flavors can be learned through repeated exposure to flavors transmitted from the maternal diet to amniotic fluid, mother’s breast milk and solid foods [32,33,34,35,36,37]. In our Mexican context, it is possible that these children had little exposure with peanuts or were not familiar to peanut paste, which could explain the lower SQ-LNS consumption compared to other studies. In fact, non-exposure to nuts was the main reason for ineligibility in our study. A second factor that may explain low acceptability of SQ-LNS is the heterogeneity of foods used for mixing the supplement. In the above-mentioned studies, the supplement was mixed with one food item (mostly maize porridge) even though a few caregivers also reported feeding the supplement alone or mixing it with other foods during the home exposure period. In our study, caregivers mixed the supplement with the food they preferred as we did not identify a universally accepted complementary food that could be recommended as a food base. During our formative research, caregivers stated their preference to mix the supplement with a variety of foods. Thus, we worked with caregivers’ preferred food that they perceived their child would like, as it would be in real conditions if the SQ-LNS were part of a nutritional program. Even though we standardized the amount of food used to mix the supplement, the variation in energy density of the different foods could have affected consumption, as has been documented in the literature [38,39]. Additionally, it is likely that the recommended food serving used in this study might have been too big for young children, especially if the supplement was offered as a snack. Caregivers were requested to mix the entire 20 g portion of SQ-LNS with 30 g of complementary food. In other studies, where acceptability was high, only 10 g of SQ-LNS was mixed with food during the observed feeding session [14] and two 10 g portions, twice a day during the home exposure period [13,14,15]. In terms of age, the children in our study were older compared to children in the acceptability studies mentioned above, as we included children from age 18–24 months old, an age group that has not been studied much and for whom it is harder to introduce new foods.

Contrary to our expectations, consumption of SQ-LNS did not increase after 14 days of repeated exposure. It is possible that the variety of foods used to mix the supplement resulted in exposure to different tastes across days. Successful repeated exposure is dependent upon repeatedly tasting the same target food over time, even in small amounts [21,23,26,40,41]. In our study, while we intended for children to be exposed to the taste of the supplement during the exposure period, it is likely that each time caregivers mixed the supplement with a different food item during the 14-day exposure period, it presented a different taste experience insufficient to result in increased acceptance. Another factor contributing to the lack of change in acceptance could be due to monotony effect; i.e., that children may have become “bored” with the peanut-like flavor of the supplement, especially since it was an unfamiliar food, or with the texture or routine of having to consume the supplement daily, as has been reported in some studies [42].

Children’s consumption of SQ-LNS did not vary significantly by supplement version at the beginning and after the 14-day repeated exposure. We observed that for both versions of the supplement, caregivers usually mixed it with sweet foods (mostly bananas, other fruits and sweetened yoghurts). Thus, a child assigned to the unsweetened SQ-LNS version may not have had the intended “non-sweet” taste experience but “sweet” instead, because of the food with which the supplement was mixed. This may have resulted in lower variation in sweetness between the two groups, explaining why we found no difference in consumption. When we adjusted for the type of food (sweet vs. unsweet) used however, we found no difference in consumption. Nevertheless, this finding has important policy implications especially in nutritional transition context where overweight and obesity are public health challenges. LNS usually contain a small amount of sugar to increase palatability. Our findings suggest that the added sugar did not seem to have any additional benefit for children’s consumption. The unsweetened version of the supplement may replace the sweetened version to achieve the same benefits, at least in countries or areas where overweight and obesity is a concern and sweet complementary foods are common. Additionally, caregivers could be encouraged to mix the supplement with fruits such as bananas as these were well accepted. Continued offering of the supplement during the 14-day home exposure shows that caregivers were willing to try to feed the supplement to the child even though consumption was low. Strategies to improve consumption of the supplement need to be explored in future studies.

We identified three distinct patterns of consumption namely low-to-medium, high and high variability. These findings are similar to what has been reported by other studies [27] suggesting that when it comes to learning to like new foods through repeated exposure, one size does not fit all and individual differences may exist in the way children respond to familiarization [43]. Additional strategies may thus need to be implemented to increase consumption in certain groups of children who respond differently to novel foods and experiences.

The results of this study should be interpreted in light of some potential limitations. We did not recommend a specific food base for mixing the supplement, which could have influenced the amount of food offered and, hence the supplement consumed. During the home exposure period, despite providing a standard spoon for mixing one scoop of selected food with the supplement, variations in the type of food base used could have made it difficult to standardize the portions offered to children across different feedings. Despite the above-mentioned limitations, we were able to estimate the amount of food consumed during the home exposure period based on the leftovers we collected from caregivers and on the information provided in the food diary. In addition, the study design had greater ecological validity in that it was conducted in home environments, which provided a realistic insight on actual child-feeding practices in our study setting and highlighted key aspects to consider when implementing supplementation programs.

In conclusion, the acceptability and consumption of unsweetened SQ-LNS was not different from the sweetened version of the supplement. Since consumption did not differ between supplement versions, we encourage the use of the unsweetened version given the potential effects that added sugar may have on weight gain especially in regions facing the double burden of malnutrition (undernutrition and overweight/obesity). Additionally, the use and promotion of the unsweetened version would align with calls to reduce added sugar in young children’s diets-particularly early exposure. More research is needed to understand the factors that drive low consumption of SQ-LNS. Children reacted differently to repeated dietary exposure (as shown by the different consumption patterns). Individual differences may exist; thus, comprehensive strategies that take into account these differences should be implemented to improve supplement consumption.

Results from the home exposure period show that children may react differently to repeated dietary exposure (as shown by the different consumption patterns). Individual differences may exist, and it is important that these are considered when implementing strategies to improve supplement consumption.

## Figures and Tables

**Table 1:** General characteristics of participants who participated in a small quantity lipid nutrient supplement feeding trial, by study group ^1^.

Variable	LNS-U *n* = 28	LNS-S *n* = 28	Total *n* = 56
**Caregiver characteristics**			
Age, years	26.4 ± 5.5	27.7 ± 7.1	27.52 ± 6.23
Parity	2.1 ± 1.2	2.0 ± 0.9	2.12 ± 1.06
BMI, kg/m^2^	25.5 ± 5.1	26.8 ± 4.5	26.2 ± 4.8
Relationship to child			
Mother	27 (96.4)	27 (96.4)	27 (96.4)
Grandmother	1 (3.6)	1 (3.6)	1 (3.6)
Marital Status			
Married, living with partner	5 (17.9)	6 (21.4)	11 (19.6)
Free union	17 (60.7)	19 (67.9)	36 (64.3)
Other ^2^	6 (21.4)	3 (10.7)	9 (16.1)
Education level completed			
None ^3^	0 (0.0)	1 (3.6)	1 (3.6)
Elementary school	7 (25.0)	7 (25.0)	14 (25.0)
Middle school	13 (46.4)	13 (46.4)	26 (46.4)
High school	5 (17.7)	3 (10.7)	8 (14.3)
Technical or college/university	3 (10.7)	4 (14.3)	7 (12.5)
Occupation			
Stays at home	21 (75.0)	26 (92.9)	47 (83.9)
Formal or informal work ^4^	7 (25.0)	2 (7.1)	9 (16.1)
**Child characteristics**			
Gestational age at birth	39.3 ± 1.4	39.4 ± 1.0	39.4 ± 1.2
Age category			
7 to 12 months	14 (50.0)	12 (42.9)	26 (46.4)
13 to 24 months	14 (50.0)	16 (57.1)	30 (53.6)
Sex			
Male	17 (60.7)	7 (25.0)	24 (42.9)
Female	11 (39.3)	21 (75.0)	32 (57.1)
Nutritional status indicators			
Length, cm	76.8 ± 6.1	74.6 ± 6.0	75.7 ± 6.1
Weight, kg	10.0 ± 1.5	9.1 ± 1.3	9.5 ± 1.4
Length for age, LAZ	−0.5 ± 1.0	−1.1 ± 1.0	−0.8 ± 1.0
Weight for age, WAZ	−0.1 ± 1.0	−0.6 ± 0.9	−0.3 ± 1.0
Weight for length, WLZ	0.3 ± 0.9	−0.1 ± 1.0	0.1 ± 1.0
BMIZ	0.3 ± 0.9	0.02 ± 0.97	0.2 ± 0.9
**Household characteristics**			
Food insecurity	23 (85.2)	21 (75.0)	44 (80.0)
Available services			
Electricity	28 (100.0)	28 (100.0)	56 (100.0)
Pipe water in home ^5^	26 (92.9)	26 (92.9)	52 (92.8)
Sanitary facility (toilet) ^6^	19 (67.8)	23 (82.1)	42 (75.0)
Bottled water	23 (85.2)	22 (78.6)	45 (81.8)

Data are presented as mean ± SD or *n* (%). ^1^ LAZ: Length for age z-score; WAZ: Weight for age z-score; WLZ: weight for length z-score; BMIZ: body mass index z-score; LNS-U = small-quantity lipid-based nutrient supplement (LNS) unsweetened; LNS-S = LNS sweetened; ^2^ divorced or single; ^3^ some elementary education; ^4^ includes domestic worker, “barista”, “comerciante”, “empleada”, “estilista”, “maestro de educacion fisica”, “negocio de quesadillas”, “renta de inflables”, “vendedora”; ^5^ complementary category: natural source, boiled water, filtered water, other; ^6^ complementary category: pit, blackhole.

**Table 2:** Children’s supplement consumption by SQ-LNS group and study stage ^1,2^.

	LNS-U *n* = 28	LNS-S *n* = 28	LNS-S vs. LNS-U
**Raw estimates**			
Initial, %	44.0 (31.4, 58.5)	34.8 (25.3, 44.0)	−9.2 (−25.5, 6.0)
Final, %	38.5 (27.8, 54.0)	31.5 (21.6, 43.0)	−7.0 (−24.5, 8.9)
Change	−5.5 (−21.0, 9.3)	−3.3 (−16.8, 13.6)	2.2 (−17.2, 24.4)
**Covariate-adjusted estimates**			
Initial, %	38.1 (26.3, 50.8)	40.1 (30.4, 50.7)	2.1 (−14.3, 18.1)
Final, %	34.4 (22.6, 47.0)	36.7 (25.4, 48.9)	2.3 (−15.1, 19.3)
Change	−3.6 (−18.1, 11.5)	−3.4 (−17.1, 12.2)	0.2 (−20.3, 18.7)

^1^ Estimates are mean percentages obtained as post-estimations from a fractional logistic regression model. Covariate-adjusted estimates are predictive margins, adjustment covariates were child sex, length for age and weight for age z-scores. Bias-corrected bootstrap 95% confidence intervals are shown in parenthesis. ^2^ LNS-U = small-quantity lipid-based nutrient supplement (LNS) unsweetened; LNS-U = LNS sweetened.

**Table 3:** Percentage of children who consumed 50% of the supplement or more, by SQ-LNS group and study stage ^1,2^.

	LNS-U *n* = 28	LNS-S *n* = 28	LNS-S vs. LNS-U
**Raw estimates**			
Initial, %	42.9 (17.9, 57.1)	39.3 (25.0, 60.7)	−3.6 (−32.1, 17.9)
Final, %	32.1 (17.9, 53.6)	25.0 (10.7, 42.9)	−7.1 (−28.6, 17.9)
Change	−10.7 (−32.1, 14.3)	−14.3 (−39.3, 10.7)	−3.6 (−32.1, 32.1)
**Covariate-adjusted estimates**			
Initial, %	32.1 (17.5, 52.3)	47.9 (28.3, 65.6)	15.7 (−10.3, 40.5)
Final, %	25.5 (12.3, 43.6)	33.4 (15.3, 52.3)	7.9 (−17.7, 31.2)
Change	−6.6 (−27.6, 14.4)	−14.5 (−40.3, 10.7)	−7.9 (−41.7, 24.8)

^1^ Estimates are mean percentages obtained as post-estimations from a logistic regression model. Covariate-adjusted estimates are predictive margins, adjustment covariates were child sex, length for age and weight for age z-scores. Bias-corrected bootstrap 95% confidence intervals are shown in parenthesis. ^2^ LNS-U = small-quantity lipid-based nutrient supplement unsweetened; LNS-U = LNS sweetened.

## Supplementary Materials

The following table is available online at https://www.mdpi.com/2072-6643/12/10/3069/s1. Figure S1: SQ-LNS nutritional information. Figure S2: individual profiles of children’s supplement consumption by consumption group. Table S1: frequency of morbidity symptoms (during the week before the interview) of the participants, by study group and study stage.
