# Supplementary material for: Effect of Added Sugar on the Consumption of A Lipid-Based Nutrient Supplement Among 7–24-Month-Old Children
Source: Nutrients. 2020 Oct 8;12(10):3069. doi: 10.3390/nu12103069 (PMC7600100; doi:10.3390/nu12103069)

Supplementary data

**Supplemental Figure 2.** Individual profiles of children’s supplement consumption by consumption group. We observed 3 consumption groups: low-medium consumption group, high consumption group and high variability group. Groups were obtained from hierarchical cluster analysis.


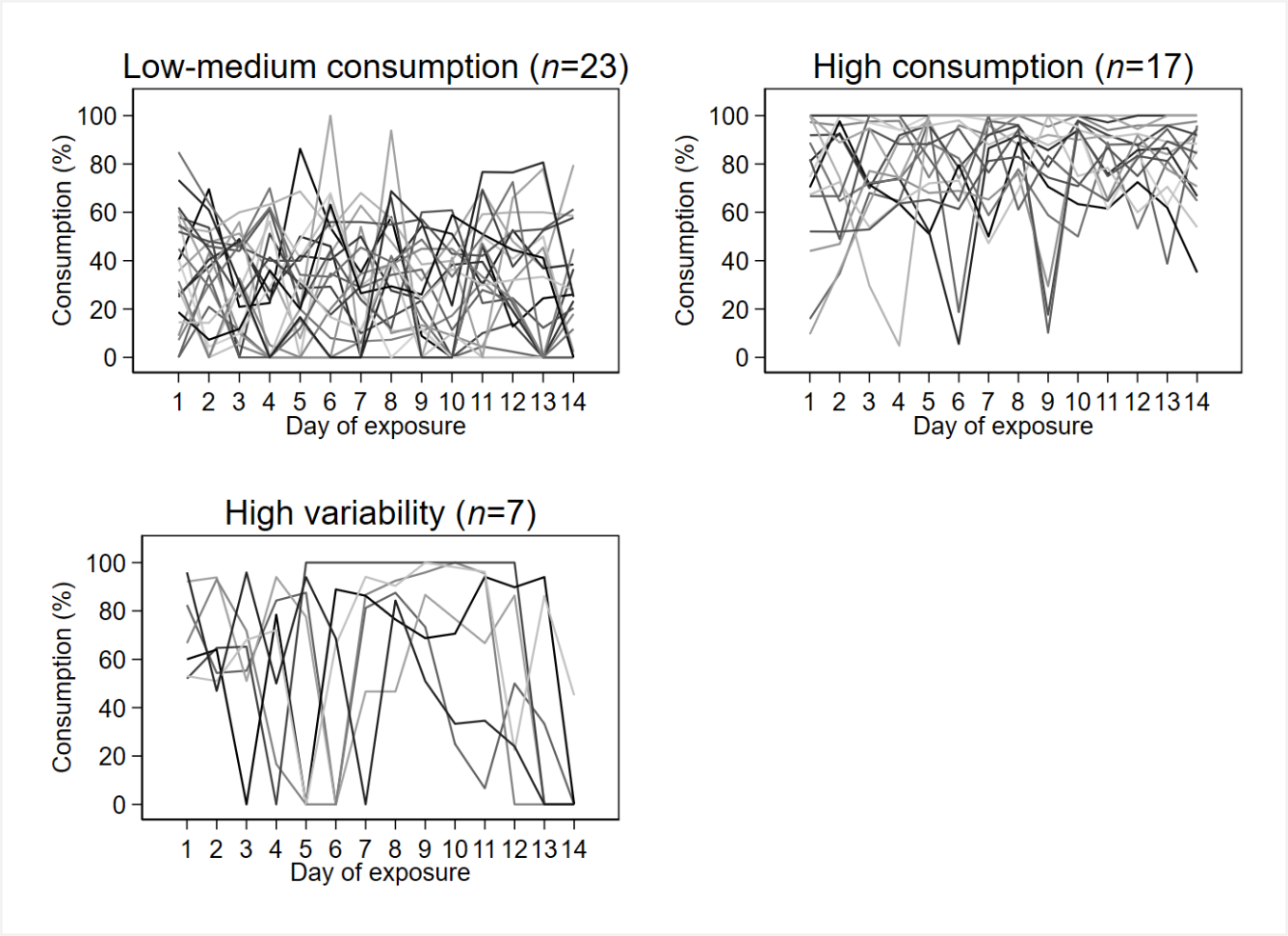

Supplement: Supplementary file 1 [file nutrients-12-03069-s001.zip › Figure S2.docx]
