# Supplementary material for: Effect of Added Sugar on the Consumption of A Lipid-Based Nutrient Supplement Among 7–24-Month-Old Children
Source: Nutrients. 2020 Oct 8;12(10):3069. doi: 10.3390/nu12103069 (PMC7600100; doi:10.3390/nu12103069)
Supplement: Supplementary file 1 [file nutrients-12-03069-s001.zip › Table S1.docx]

| **Supplemental Table 1:** Frequency of morbidity symptoms (during the week before the interview) of the participants, by study group and study stage ^1,2^ | | | | |
| --- | --- | --- | --- | --- |
|  | Initial exposure | | Final exposure | |
| Symptom | LNS-U  n=28 | LNS-S  n=28 | LNS-U  n=27 | LNS-S  n=28 |
| Diarrhea (%) | 2 (7.1) | 5 (17.9) | 5 (18.5) | 3 (10.7) |
| Vomit (%) | 2 (7.1) | 2 (7.1) | 3 (11.1) | 3 (10.7) |
| Cough (%) | 11 (39.3) | 11 (39.3) | 7 (25.9) | 11 (39.3) |
| Mucus (%) | 6 (21.4) | 9 (32.1) | 7 (25.9) | 8 (28.6) |
| Difficulty breathing (%) | 1 (3.6) | 2 (7.1) | 1 (3.7) | 1 (3.6) |
| Rapid breathing (%) | 1 (3.6) | 1 (3.6) | 0 (0.0) | 0 (0.0) |

^1^LNS-U = Small-Quantity Lipid-based Nutrient Supplement (LNS) unsweetened; LNS-U = LNS sweetened.

^2^No statistically differences were found between study groups at both initial and final exposure (p>0.39; Fisher exact test for contingency tables).
