# Supplementary figures and images for: Effect of Added Sugar on the Consumption of A Lipid-Based Nutrient Supplement Among 7–24-Month-Old Children
Source: Nutrients. 2020 Oct 8;12(10):3069. doi: 10.3390/nu12103069 (PMC7600100; doi:10.3390/nu12103069)

**Supplemental Figure 1.** SQ-LNS Nutritional information

**
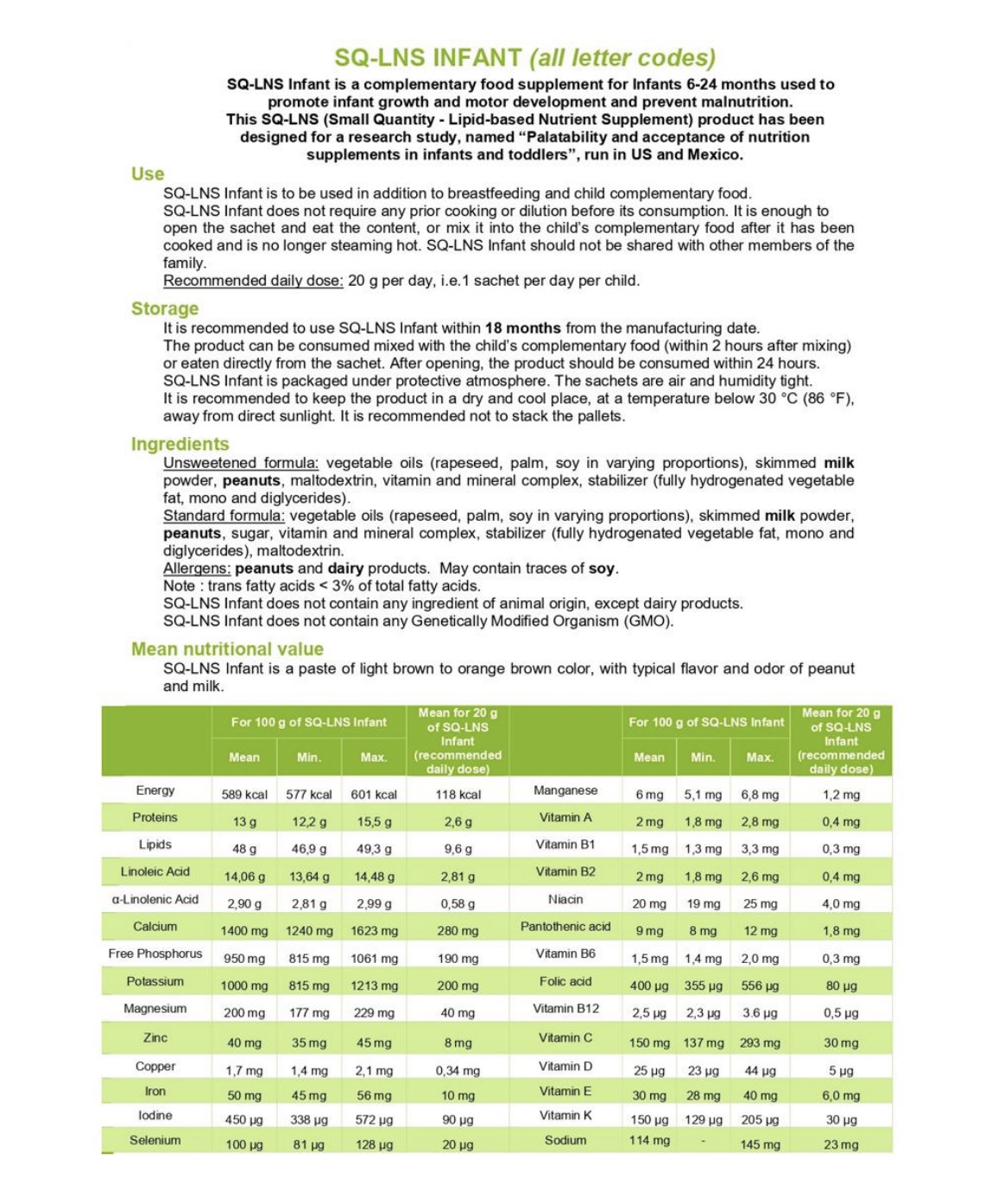
**

Supplement: Supplementary file 1 [file nutrients-12-03069-s001.zip › Figure S1.docx]
